# Calciphylaxis as A Rare Cause of A Chronic Wound in An 83-Year-Old Woman

**DOI:** 10.3390/geriatrics4010028

**Published:** 2019-03-18

**Authors:** Stefan Dörr, Gregor Weisser, Ralf Lobmann

**Affiliations:** Department of Endocrinology, Diabetology and Geriatrics, Stuttgart General Hospital, 70374 Bad Cannstatt, Prießnitzweg 24, Germany; g.weisser@klinikum-stuttgart.de

**Keywords:** calciphylaxis, Martorell hypertensive ischemic leg ulcer, HYTILU, chronic wound, wound healing, phenprocoumon, calcium–phosphate balance, vitamin D

## Abstract

Chronic wounds are common in elderly patients, and the majority of them are caused by vascular diseases, such as peripheral arterial occlusive disease (PAD) or chronic venous insufficiency. Because of typical signs, these diseases can be usually easily differentiated. However, 10% of chronic wounds are caused by specific rare diseases, such as vasculitis, specific infections, skin cancer, or calciphylaxis. Calciphylaxis is a rare cause of chronic wounds, and it is usually found in patients with end-stage renal disease. In this paper, we describe the case of an 83-year-old woman with a chronic ulcer of the lower leg caused by calciphylaxis.

## 1. Introduction

Calciphylaxis of the distal and proximal pattern, Martorell hypertensive ischemic leg ulcer (HYTILU), and “calciphylaxis with normal renal and parathyroid function” (CANREPAF) are probably pathophysiologically interlinked because of common risk factors and mutual clinical pattern, i.e., necrotizing livedo and painful skin infarctions at typical locations. In histopathology subcutaneous stenotic arteriolosclerosis with thickening of the vessel wall due to hyperplasia of the smooth muscle layer and/or intimal hyperplasia are found, as well as miniaturizing Mönckeberg medial calcinosis. Internal organs, such as kidney or stomach, may also be affected [1].

Calciphylaxis normally besets patients with chronic renal insufficiency or after successful kidney transplantation. The distal form (localized at any site of the leg, predominantly laterodorsal above the Achilles tendon) is linked with a better prognosis than the proximal form (localized at inner thighs, abdominal apron, and upper arms) [2].

The extremely painful HYTILU, first described in 1940 by Haxthausen, has its typical location on the dorsolateral leg above the Achilles tendon [3]. They are also distinguished by wall thickening in the arterioles. Most patients are older than 60 years and have had arterial hypertension for many years, mostly well controlled. In addition, 60% have type 2 diabetes, and 50% have classic peripheral arterial occlusive disease [4].

Ulcers due to CANREPAF consistently affect morbidly obese people suffering from type 2 diabetes and arterial hypertension [5]. The ulcers typically occur in an area where the fatty tissue is outstandingly thick (inner thigh, abdominal apron, breasts, upper arms). CANREPAF and HYTILU appear to be two variants of the same disease, corresponding to calciphylaxis of distal or proximal pattern [4]. Clinically and histopathologically, CANREPAF is indistinguishable from calciphylaxis of proximal pattern but for the difference of a normal kidney function in these patients [5].

In 2010, Hafner [1] postulated that four major risk factors cause this ischemic arteriolosklerosis: (I) hypertension as the driving risk factor, (II) diabetes mellitus (type 1 or 2), (III) secondary hyperparathyroidism, and (IV) oral anticoagulation with vitamin K antagonists because the α_2_-Heremans–Schmid glycoprotein (AHSG, synonym matrix Gla protein) is a potent inhibitor of pathological calcification and requires vitamin K-dependent γ-carboxylation.

Major differential diagnoses for the four entities named above are pyoderma gangrenosum and necrotizing vasculitis.

## 2. Patient’s Characteristics

All subjects gave their informed consent for inclusion before they participated in the study.

The 83-year-old woman suffered from a painful ulcer of the lower leg for about six months. At the beginning, she noticed a raised swelling at the shinbone, which developed into an ulcer within a few weeks without any tendency of healing. She did not remember any trauma in this area. Additionally, she suffered from breast cancer metastasized into bone (jawbone), and she was treated with phenprocoumon until a year ago because of atrial fibrillation before the treatment was switched to apixaban. Furthermore, peripheral arterial occlusive disease and arterial hypertension were known. She had osteoporosis and was malnourished after a two-thirds resection of the stomach (history of gastric ulcers) and had difficulties in chewing due to the metastasis of the jawbone.

Because of the evolving ulcer, she was previously treated with antibiotics in our Department of Dermatology, and a vasculitis was ruled out via biopsy. After an initial improvement during the inpatient treatment, she noticed a deterioration once again at home and was finally admitted to our department.

**Figure 1:**
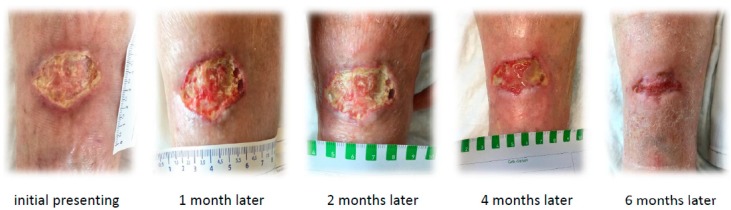
Initial wound finding and course of wound healing.

We first saw the 83-year-old, gaunt lady with an ulcer of the shinbone measuring 35 × 32 mm. The wound margin was bulging, macerated, and with livid discoloration. The wound bed was covered with fat tissue necrosis and was bony hard. The surrounding wound was swollen and painful (Figure 1).

Laboratory chemical examinations (Table 1) showed a mildly reduced renal function, slightly increased albumin-corrected calcium and parathyroid hormone, and a deficiency of vitamin D and albumin.

## 3. Therapy

**Figure 2:**
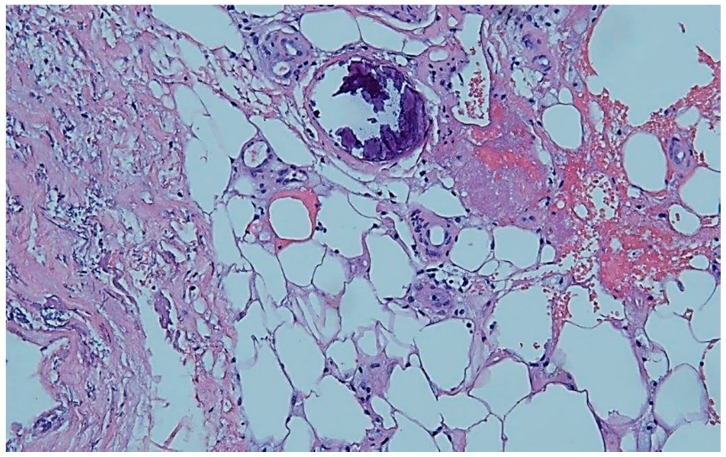
Histopathology: Typical calcification of a small vessel in the upper corium. By courtesy and permission of Prof. Dr. P. von den Driesch, Dept. of Dermatology, Stuttgart General Hospital.

Despite use of a good wound therapy, including an attempt to use negative-pressure wound therapy, we could achieve only a poor improvement. This led us to the decision to take a histological sample. Subepithelial increase of small vessels with inflammatory alteration and distinct hemosiderosis were found in the biopsy. Subsequently, a typical calcification of a small vessel was described in the upper corium, primarily pointing to calciphylaxis (Figure 2). There was no evidence of a local vasculitis or a metastasis of the known breast cancer.

The moderately elevated parathyroid hormone (PTH), in combination with a normal plasma level of phosphate and slightly elevated calcium, was interpreted as secondary in the course of a deficiency of cholecalciferol (vitamin D). Vitamin K level was not tested and, unfortunately, prothrombin time was not usable because of apixaban medication.

We finally established a treatment with vitamins K and D. Bisphosphonates were also added due to a recommendation by our oncologist concerning the osteoporosis and metastasis in the jawbone. In combination with wound therapy with a fine-pore polyurethane foam and intermittent adequate but restrained debridement of the wound base, we could finally record a considerable amelioration in wound healing over approximately six months (Figure 1). To alleviate pain, we successfully used an ibuprofen containing fine-pore polyurethane foam in addition to an opioid-based oral pain therapy. During treatment in our outpatient care, there was, unfortunately, no further control of serum calcium, phosphate, or parathyroid hormone. Because of the marked improvement in wound healing in this case of a non-life-threatening calciphylaxis, we avoided infused sodium thiosulfate.

## 4. Discussion

Calciphylaxis is a rare disease that is primarily seen in patients with advanced chronic kidney disease or after kidney transplantation. In addition to the four major risk factors—hypertension, diabetes mellitus (type 1 or 2), secondary hyperparathyroidism, and oral anticoagulation with vitamin K antagonists—several other risk factors like female gender, obesity, protein deficiency within malnutrition, and chronic inflammation have been proposed [1,6,7].

It was first described in 1960 by pathophysiologist Prof. Hans Selye [8], who could provoke metastatic calcification in animals in a two-step manner. In the preparation stage, called “sensitization”, he applied high doses of vitamin D or parathyroid hormone, followed by the intravenous or intraperitoneal application of iron salts or protein, the ”challenge” that led to the calcification. However, he did not demonstrate atherosclerosis with medial calcification. Therefore, for a long time, the cause for calciphylaxis was seen as the raised calcium x phosphate product in patients with chronic kidney disease and the compensating secondary hyperparathyroidism. Due to an increased production of the parathyroid hormone, there is an increase in bone remodeling and loss [6,7,8].

Calciphylaxis and HYTILU share a common clinical appearance and pathophysiology. Calciphylaxis, in its distal form, and HYTILU typically occur on the laterodorsal leg above the Achilles tendon. It is quite possible that our patient suffered from HYTILU because of pain at, approximately the typical location, the absence of advanced renal insufficiency, and existing comorbidities, such as arterial hypertension and peripheral arterial occlusive disease. Our biopsy yielded a characteristic calcification of a small vessel, consistent with miniaturized Mönckeberg sclerosis, but the description was not more precise. Calciphylaxis of proximal pattern and CANREPAF could be excluded because they are typically found at inner thighs, abdominal apron, breasts, and upper arms. CANREPAF predominantly attacks the morbidly obese [1,4,9].

The therapeutic options are still limited. As the ulcers commonly tend to be a bacterial superinfection, antibacterial or even antibiotic treatment is often required [8]. First and foremost, the aim is to normalize calcium and phosphate plasma levels in cases with a disorder of calcium–phosphate balance by tweaking the dialysis modalities and adjunctive therapy (intensification of dialysis, lowering calcium concentration in the dialysate, and high-dose administration of phosphate binders). Stopping a high-dose substitute of vitamin D should also be considered. Patients with hyperfunction of the parathyroid gland should be considered for the possibility of an immediate parathyroidectomy or the use of calcimimetica. The latter ones result in functional parathyroidectomy [8]. As we could verify a deficiency of vitamin D, we administered 25-hydroxy vitamin D.

The α_2_-Heremans–Schmid glycoprotein is a potent inhibitor of pathological calcification, which requires vitamin-K-dependent γ-carboxylation. Therefore, there is a strong recommendation to stop treatment with vitamin K antagonists and to substitute vitamin K orally. For this reason, we started treatment with vitamin K. 

To allow the HYTILU to heal simply by getting the hypertension under control is short-sighted. Usually, arterial hypertension is already well controlled, and the arteriolopathy is not functional but morphologically fixed [1]. 

A number of work groups have empirically published that the treatment for skin infarction due to ischemic arteriolosclerosis is primarily surgical. Excision of the necrotic tissue and early covering with a skin graft should be rapid and effective [10]. In our case, the wound base appeared to be not quite vital and suitable for a skin graft, and so we neglected it. Instead, we applied negative-pressure wound therapy to activate the wound base but with poor success. Finally, we chose an ibuprofen containing fine-pore polyurethane foam for wound management. Fine-pore polyurethane foams offer warm and humid wound conditions for granulation tissue and cover the wound in a nonocclusive manner.

Successful treatment with bisphosphonates or infusion of sodium thiosulfate has been described, but it should only be done in centers with experience in treating calciphylaxis. We avoided treatment with sodium thiosulfate because of the improvement in wound healing and because it was a non-life-threatening case. Sodium thiosulfate has been found to bind calcium in patients with calciphylaxis. It is effective in patients with classical calciphylaxis as well as CANREPAF [11]. Common side effects are nausea and vomiting due to anion gap acidosis. A treatment with bosentan also seems to be effective, as Sillera-Herrera reported [12]. For further information about diagnosis, treatment options, or current studies, see www.calciphylaxis.net, the platform of calciphylaxis network.

Differential diagnoses are pyoderma gangrenosum and necrotizing vasculitis. There was no evidence in the histological sample for vasculitis, and our patient had no typical underlying neutrophilic disorders, such as inflammatory bowel disease, rheumatoid arthritis, or hematoproliferative disorder.

## 5. Summary

In this work, we have described the case of an 83-year-old woman with a chronic, painful ulcer of the lower limb. Because of a calcification of the wound base and absence of wound healing, we decided to take a biopsy, which revealed the typical calcification of a small vessel (miniaturized Mönckeberg sclerosis) found in calciphylaxis or HYTILU. We therefore initiated a treatment with vitamin K substitute, bisphosphonates, and a substitute for vitamin D deficiency. Our patient showed a mildly impaired renal function but appeared with various risk factors for calciphylaxis, such as female gender, protein deficiency within malnutrition, and a disorder of the calcium–phosphate household. A probable cue in this case might also be the long-term treatment with phenprocoumon due to atrial fibrillation in former years. The history of breast cancer may have contributed, as Anderson had reported histologically proven calciphylaxis as being associated with metastatic cholangiocarcinoma without end-stage renal disease in a post-mortem study [13].

Under our medical treatment and with good wound management using a fine-pore polyurethane foam, we could achieve a significant improvement in wound healing at the end. 

This case shows that a small proportion of ulcers in elderly people have a specific cause but that it is necessary to think of taking a histological sample if the wound characteristics give cause for reconsideration.

## Figures and Tables

**Table 1:** Blood results.

Parameter	Unit	Result	Normal Range
Hb	g/dL	10.0 (−)	12–16
Leucocytes	Tsd/µL	4.3	4–10
Prothrombin time	%	75 %	70–100
Creatinin	mg/dL	0.8	0.5–0.9
GFR (CKD-EPI formula)	mL/min	68	60–180
Calcium	mmol/L	2.4	2.0–2.5
Calcium, adjusted	mmol/L	2.6 (+)	
Albumin	g/L	31.6 (−)	34–50
Phosphate	mg/dL	2.5	2.3–4.7
Parathyroid hormone	ng/L	132 (+)	15–65
25-OH cholecalciferol	µg/L	14 (−)	> 30 μg/L
